# Impact of cognitive behavior therapy on osteoarthritis-associated pain, insomnia, depression, fatigue, and physical function in patients with knee/hip osteoarthritis: A systematic review and meta-analysis of randomized controlled trials

**DOI:** 10.3389/fmed.2022.1083095

**Published:** 2023-01-06

**Authors:** Hong-Min Lin, Pei-Shan Hsieh, Nai-Ching Chen, Chiung-Huei Tsai, Wen-Fu Kuo, Ying-Li Lee, Kuo-Chuan Hung

**Affiliations:** ^1^Department of Family Medicine, Chi Mei Medical Center, Tainan, Taiwan; ^2^Department of Nursing, Chi Mei Medical Center, Tainan, Taiwan; ^3^Department of Nursing, Chang Jung Christian University, Tainan, Taiwan; ^4^School of Medicine, College of Medicine, National Sun Yat-sen University, Kaohsiung City, Taiwan; ^5^Department of Anesthesiology, Chi Mei Medical Center, Tainan, Taiwan

**Keywords:** cognitive behavior therapy, osteoarthritis, meta-analysis, pain, insomnia

## Abstract

**Background:**

This meta-analysis aimed at evaluating the efficacy of cognitive behavior therapy (CBT) against osteoarthritis-associated symptoms in patients with knee/hip osteoarthritis.

**Methods:**

Medline, PubMed, Cochrane Library, and EMBASE databases were searched from inception to July 2022 to identify randomized controlled trials (RCTs) comparing the efficacy of CBT with other treatment approaches in adults with confirmed knee/hip osteoarthritis. The pain intensity (primary outcome) and the secondary outcomes including insomnia severity, sleep efficiency, physical function as well as the severity of depression and fatigue were assessed at two time points (i.e., immediately after treatment and during the follow-up period). The effect size is expressed as standardized mean difference (SMD) with SMDs of < 0.2, 0.2–0.5, and 0.5–0.8, and > 0.8 representing negligible, small, medium, and large effect sizes, respectively.

**Results:**

Fifteen RCTs were included for analysis. Immediately after CBT intervention, meta-analysis showed similar treatment effect in pain severity [SMD = –0.46, 95% confidence interval (CI): –0.95 to 0.04, 11 studies, 1557 participants] and other symptoms including depression (SMD = –0.26, 95% CI: –0.58 to 0.06, five studies, 735 participants), fatigue (SMD = –2.44, 95% CI:–6.53 to 1.65, two RCTs, 511 participants), and physical function (SMD = –0.11, 95% CI:–0.25 to 0.02, five RCTs, 720 participants) between CBT and control groups, while there was an improvement in insomnia severity (SMD = –0.65, 95% CI: –1.06 to –0.24, four RCTs, 639 participants, medium treatment effect) and sleep efficiency (SMD = 0.32, 95% CI: 0.04 to 0.59, three RCTs, 352 patients, small treatment effect). During follow-up, CBT improved pain severity (SMD = –0.52, 95% CI: –1.03 to –0.01, eight studies, 1447 participants, medium treatment effect), insomnia (SMD = –0.43, 95% CI: –0.85 to –0.01, three RCTs, 571 participants, small treatment effect), and depression (SMD = –0.39, 95% CI: –0.59 to –0.18, four RCTs, 791 participants, small treatment effect). Nevertheless, sleep efficiency, fatigue, and physical function were not improved in the follow-up period.

**Conclusion:**

Our results may suggest the durability of CBT-associated treatment benefits, supporting its role as a potential promising alternative or complementary intervention for patients with knee/hip osteoarthritis, especially against pain and insomnia. Future large-scale investigations are warranted to verify our findings.

**Systematic review registration:**

[https://www.crd.york.ac.uk/prospero/], identifier [CRD42022331165].

## 1. Introduction

Osteoarthritis, the most common form of arthritis, is one of the leading causes of musculoskeletal pain and disability worldwide (1). Joint pain in most patients with osteoarthritis is associated with reduced self-efficacy, depressed mood, and impaired sleep (2). Such complex psychosocial influences result in a varying range of functional limitations, psychological dysfunction, and impact on quality of life (2). Other psychosocial factors can also predict a higher degree of pain in osteoarthritis patients, including anxiety, depression, pain catastrophizing, and social isolation (3–5). Moreover, individuals with osteoarthritis also commonly report comorbid insomnia, the most prevalent form of sleep disturbance associated with chronic pain (6). Sleep deprivation is considered to interfere with cognitive function, emotion regulation, and pain sensation (7, 8), thereby aggravating their chronic pain condition (6, 9). Therefore, a comprehensive plan for the management of osteoarthritis should encompass educational, behavioral, psycho-social, and physical interventions, in addition to pharmacological treatment (10).

There is a body of literature showing the beneficial effects of non-pharmacologic treatments on pain reduction in patients with mild to moderate osteoarthritis, including pain education, manual therapy, and therapeutic exercise with or without combining with dry needling (11–14). Cognitive behavioral therapy (CBT) is a structural, time-limited, goal-oriented psychological treatment that incorporates behavioral strategies and cognitive processes for specific problems (15). Although it was primarily developed as a therapy for depression, its efficacy for various psychological (e.g., anxiety disorders, personality disorders, and eating disorders) and chronic (e.g., insomnia, chronic spinal pain, and osteoarthritis) conditions has also been reported (15–20). Therefore, several different CBT approaches have been developed, such as CBT for insomnia (CBT-I) and pain coping skills training (PCST). In patients with osteoarthritis, there have been previous meta-analytic studies showing the effectiveness of cognitive behavioral therapies for improving arthritic pain, sleep quality (e.g., insomnia severity and sleep efficiency), and psychosocial outcomes (e.g., self-efficacy, depression, and psychological distress) (20–24). On the other hand, the efficacy of CBT and the sustainability of its treatment benefits as an additive therapy for enhancing exercise adherence remain controversial in patients with osteoarthritis (24, 25). Moreover, the level of evidence was blemished by the inclusion of a limited number of studies or combination with other interventions (e.g., exercise) (21–23). To focus on the treatment effects of CBT, we excluded studies that combined CBT with other active treatments (e.g., exercise), which have been shown to be effective against osteoarthritis-associated psychosomatic comorbidities in published clinical guidelines (10, 26). Therefore, the aim of this systematic review is to investigate the efficacy of CBT without other combined treatment against osteoarthritis-associated symptoms, namely, pain, insomnia, physical function, and other psychological factors, in both the post-intervention and follow-up periods.

## 2. Methods

The findings of this meta-analysis were reported in accordance with the recommendations of the PRISMA statement (27). The protocol of this study was registered with the International Prospective Register of Systematic Reviews (CRD42022331165).

### 2.1. Search strategies

Three databases including the MEDLINE (Ovid) (from 1946 to July 2022), EMBASE (Ovid) (from 1974 to July 2022), and Cochrane library (from 1947 to July 2022) were searched. The reference lists of the acquired articles and Google scholar were also manually searched for potentially eligible studies. To access all possible MeSH terms, the following search terms were applied: “osteoarthritis” OR “osteoarthritis, hip” OR “osteoarthritis, knee.” The second group keywords were “cognitive behavioral therapy” OR “pain coping skill training” OR “behavior graded activity.” The two groups were combined using “AND.” Only randomized controlled trials (RCTs) published in English were considered eligible. We applied no restrictions to publication date and size of the sample. The information regarding the search strategies used is available in Supplementary Table 1.

### 2.2. Inclusion and exclusion criteria

Studies were eligible for review with reference to the following criteria: (1) Participants: adults with confirmed diagnosis of knee or/and hip osteoarthritis based on physician evaluation, radiographic evidence combined with self-reported osteoarthritis-related symptoms. Participants who underwent joint replacement surgery for knee/hip OA were excluded; (2) Intervention: any treatment which fulfilled the criteria for CBT regardless of its approach (e.g., face-to-face or internet-based) was eligible. We only included trials that adopted a combined cognitive-behavioral intervention model; (3) Control: interventions including routine care, education-only control, attention control, or no treatment were considered eligible for serving as controls; (4) Outcome measures: osteoarthritis-associated symptoms (e.g., pain severity, insomnia).

Exclusion criteria were: (1) studies which combined CBTs with any other active therapy, including exercise or physical therapy; (2) those using mindfulness- or hypnosis-based components as active treatments or as part of CBTs; (3) those without details about outcomes, and (4) studies not published as full-length original research papers such as letters, abstracts, reviews, case reports, or other forms of publication.

### 2.3. Studies selection and data collection

Two independent reviewers selected the eligible studies by examining their titles and abstracts based on the inclusion and exclusion criteria. An independent third reviewer resolved any disagreements that arose between the two reviewers. A specially designed data extraction tool (using a set of templates) was used by the two authors for independent data extraction from the studies. In the case that disagreements could not be settled through discussion, a third review author was consulted.

### 2.4. Outcomes and definitions

The primary outcome was the degree of pain relief following CBT intervention. The secondary outcomes included insomnia severity, sleep efficiency, physical function, depression, and fatigue. The definitions of primary and secondary outcome assessments were in accordance with those adopted in individual studies regardless of the tools being applied [e.g., Visual Analogue Scale (VAS) or Western Ontario and McMaster Universities Osteoarthritis Index (WOMAC) pain subscale for pain severity; Insomnia Severity Index or Wake After Sleep Onset Diary for insomnia]. In the current meta-analysis, we evaluated the efficacy of CBT against the related symptoms at two time points, namely, immediately after treatment and during the follow-up period. If data from multiple follow-up time points were available in a study, we selected the latest time point for data extraction.

### 2.5. Risk of bias assessment and certainty of evidence

Two independent reviewers assessed the risk of bias of each included trial. Disagreements were resolved by discussion till a consensus was reached, otherwise a third review author acted as arbiter. According to RoB 2.0 (28), we assessed the risk of bias for each study based on the key criteria: randomization process, deviation from intended intervention, missing outcome date, measurement of the outcome, selection of the report result, and overall bias. We judged the risk of bias according to each of the domains as: low, some concerns, and high.

The overall certainty of evidence for each outcome was investigated using the Grading of Recommendations Assessment, Development and Evaluation (GRADE) framework (29). Disagreements regarding overall certainty of evidence were resolved by discussion.

### 2.6. Data analysis

Due to a variation in assessment scales for the same outcome, the effect sizes are expressed as standardized MD (SMD) including 95% confidence interval (CI) in the current study. The effect sizes were considered minimal, small, medium, and large for SMD with values of < 0.2, 0.2–0.5, 0.5–0.8, and > 0.8, respectively (30). Meta-analysis of the data was performed if more than two trials that reported the same outcome of interest. *I*-squared (*I*^2^) test was used to assess heterogeneity among the RCTs with thresholds being set at > 50% for defining significant heterogeneity as previously reported (31, 32). Assuming a high heterogeneity across the included trials, we applied *a priori* a random-effects model regardless of the outcomes of statistical heterogeneity. The likelihood of publication bias was investigated by inspection of a funnel plot when there are ten or more studies reporting the same outcome. To evaluate the influence of an individual study on the pooled results, leave-one-out sensitivity analysis was performed (31). Meta-regression was performed to identify the origin of heterogeneity by using the comprehensive Meta-Analysis (CMA) V3 software (Biostat, Englewood, NJ, USA). The following covariates were included for meta-regression analysis, including age, proportion of female, sample size, and follow-up time. Other analyses were conducted with the Cochrane Review Manager (RevMan 5.3; Copenhagen: The Nordic Cochrane Center, The Cochrane Collaboration, 2014). Two-tailed statistical tests with a significant level set at *p* < 0.05 were applied.

## 3. Results

### 3.1. Characteristics and quality of studies

**FIGURE 1:**
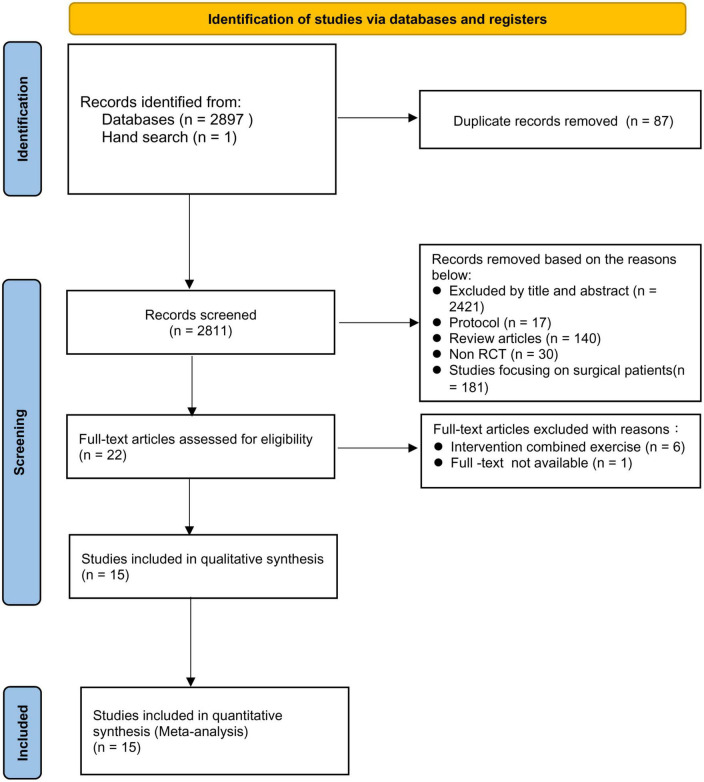
Screening process for study selection.

A total of 2,898 relevant records were identified through electronic database and manual literature search. After removal of duplicate records, 2,821 records were available. After eliminating 2,799 articles after screening based on title and abstract as well as exclusion criteria, 22 studies were eligible for full-text screening. Seven studies were further excluded because of a combination with exercise (*n* = 6) in their intervention groups and unavailability of full-text (*n* = 1). Finally, 15 RCTs published between 1990 and 2021 were included in this systematic review and meta-analysis (33–47). A detailed flow diagram is presented in Figure 1.

Characteristics of the included studies are summarized in Table 1. In our systematic review, a total of 2,864 patients were included. The mean age of the participants was between 57.9 and 73.1 years, while one study did not provide relevant details (46). Of the 15 analyzed studies, seven recruited only patients with knee osteoarthritis (33, 36, 40, 42–44, 46), four included patients with knee/hip osteoarthritis (34, 38, 41, 45), and four did not mention the location of osteoarthritis (35, 37, 39, 47). The sample sizes ranged from 30 to 367. The methods for diagnosis of osteoarthritis are shown in Supplementary Table 2. The diagnosis of osteoarthritis was confirmed by physician or radiographic findings combined with osteoarthritis-related symptoms in all studies (33–47). The types of interventions included CBT for insomnia (CBT-I) (four RCTs) (35, 42, 43, 47), CBT for pain (CBT-P)/CBT for pain and insomnia (CBT-PI) (two RCTs) (37, 39), CBT for depression (one RCT) (44), CBT (two RCTs) (40, 46), pain coping skills training (PCST) (five RCTs) (33, 36, 38, 41, 45), and behavioral graded activity (BGA) (one RCT) (34). The CBT protocol and components of the included studies are detailed in Supplementary Table 3. Delivery of interventions included face-to-face approach (within a group or individually) in 11 studies, telephone-approach in four studies, and internet-based approach in two studies (Supplementary Table 3). The duration of intervention sessions varied from 6 to 24 weeks, with a frequency ranging from every week to every 2 weeks (Supplementary Table 3).

**TABLE 1:** Characteristics of included studies (*n* = 15).

References	Female (%)	Age (year)	*N*	Type of OA (knee/hip/both)	Intervention (*n*)	Control (*n*)	Follow-up time	Outcome	Country
Keefe et al. (33)	71%	64	99	100%/0/0	PCST (32)/Arthritis education (36)	Standard care control (31)	Post treatment	^a^^,^ ^b^	USA
Veenhof et al. (34)	78%	64.8	200	65%/40%/10%	BGA (97)	Usual care (103)	65 weeks	^a^^,^ ^b^	Netherlands
Vitiello et al. (35)	88.2%	67.7	51	NA	CBT-I (23)	SMW (28)	48 weeks	^a^^,^ ^c^^,^ ^d^	USA
Somers et al. (36)	79%	57.9	232	100%/0/0	PCST + BWM (62)/PCST-only (60)/BWM-only (59)	Standard care control (51)	48 weeks	^a^^,^ ^b^	USA
Vitiello et al. (37)	78.5%	73.1	352	NA	CBT-P (117)/CBT-PI (113)	EOC (122)	36 weeks	^a^^,^ ^c^	USA
McCurry et al. (39)	78.2%	73.1	367	NA	CBT-P (122)/CBT-PI (122)	EOC (123)	72 weeks	^a^^,^ ^c^	USA
Broderick et al. (38)	76.65%	67.2	257	77.4%/22.6%/0	PCST (129)	Usual care (128)	48 weeks	^a^^,^ ^b^^,^ ^d^^,^ ^e^	USA
Helminen et al. (40)	69%	63.6	111	100%/0/0	CBT + GP care (55)	GP care (56)	48 weeks	^a^^,^ ^d^	Finland
Smith et al. (42)	79%	59.4	100	100%/0/0	CBT-I (50)	BD (50)	24 weeks	^a^^,^ ^c^	USA
Rini et al. (41)	81%	67.6	113	35.3%/12.3%/52.2%	Internet PCST (58)	Assessment only (55)	Post treatment	^a^^,^ ^b^	USA
Heffner et al. (43)	60%	61	30	100%/0/0	CBT-I (16)	No treatment (14)	Post treatment	^a^^,^ ^c^	USA
O’Moore et al. (44)	80%	62	77	100%/0/0	OA TAU + internet CBT for depression (49)	OA TAU (28)	12 weeks	^a^^,^ ^d^	Australia
Allen et al. (45)	49.2%	59	248	79.3%/10.7%/10%	PCST (124)	Usual care (124)	36 weeks	^a^^,^ ^b^^,^ ^d^	USA
Foo et al. (46)	82.7%	NA	300	100%/0/0	CBT (150)	Routine care (150)	24 weeks	^a^^,^ ^b^^,^ ^d^	Malaysia
McCurry et al. (47)	74.6%	70.2	327	NA	CBT-I (163)	EOC (164)	48 weeks	^a^^,^ ^c^^,^ ^d^^,^ ^e^	USA

^a^Pain.

^b^Physical function.

^c^Sleep efficiency.

^d^Depression.

^e^Fatigue.

BD, behavior desensitization; BGA, behavior graded activity; BWM, lifestyle behavioral weight management; CBT, cognitive behavior therapy; CBT-I, cognitive behavior therapy for insomnia; CBT-P, cognitive behavior therapy for pain; CBT-PI, cognitive behavior therapy for pain and insomnia; EOC, education-only control; GP, general practitioner; OA, osteoarthritis; PCST, pain coping skills training; SMW, attention-control stress management and wellness; TAU, treatment as usual.

**FIGURE 2:**
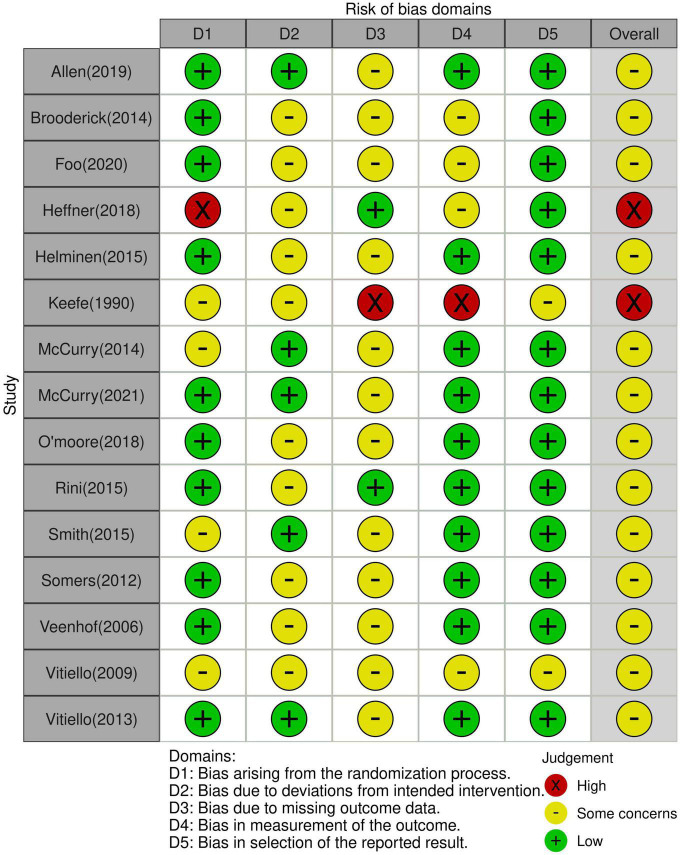
Risk of bias summary of individual studies based on reviewers’ judgment.

The risks of bias of the included studies are summarized in Figure 2. The risks of bias were mostly related to the randomization process, deviations from intended intervention, and missing outcome data. The overall risk of bias was judged to be of some concerns in two studies and high in the other 13 studies. The overall certainty of evidence was demonstrated in Supplementary Table 4. The overall certainty of evidence was graded as high in two outcomes (i.e., depression symptom during the follow-up period and physical function immediately after treatment), while other outcomes were considered as moderated.

### 3.2. Results

#### 3.2.1. Primary outcome: Impact of CBT on pain severity

**FIGURE 3:**
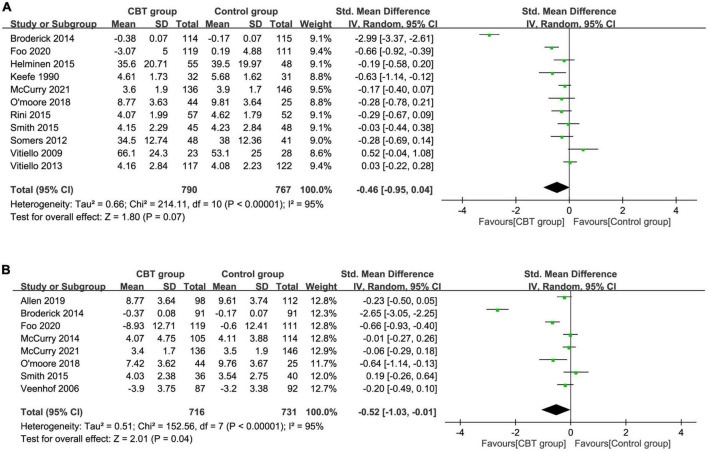
Forest plot comparing pain severity **(A)** immediately after treatment and **(B)** during follow-up period between the cognitive behavior therapy (CBT) and control groups. Std, standardized; iv, inverse variance; CI, confidence interval.

Analysis of 11 RCTs that reported immediate post-treatment pain relief demonstrated comparable treatment effect on pain severity between the CBT and control groups (SMD = –0.46, 95% CI: –0.95 to 0.04, *P* = 0.07, *I*^2^ = 95%, 11 studies, 1557 patients) (Figure 3A) (33, 35–38, 40–42, 44, 46, 47) with an unstable result on sensitivity analysis. During post-treatment follow-up, pooled result from a total of eight RCTs revealed a medium treatment effect of CBT on pain severity (SMD = –0.52, 95% CI: –1.03 to –0.01, *P* = 0.04, *I*^2^ = 95%, eight studies, 1447 patients) (Figure 3B) (34, 38, 39, 42, 44–47) with an unstable finding on sensitivity analysis. An inspection of the funnel plot showed a low risk of publication bias on pain severity immediately after treatment (Supplementary Figure 1).

#### 3.2.2. Secondary outcomes: Insomnia severity

**FIGURE 4:**
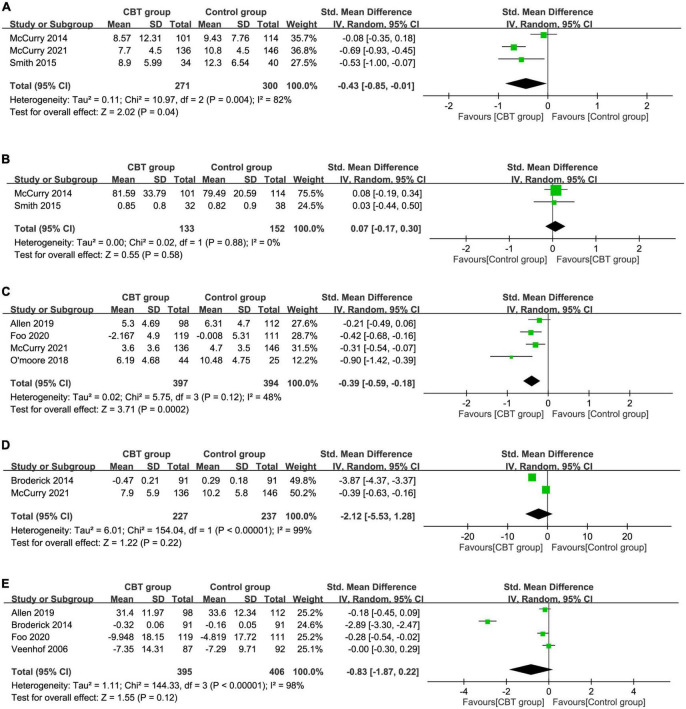
Forest plot comparing **(A)** insomnia severity, **(B)** sleep efficiency, **(C)** depression severity, **(D)** fatigue severity, and **(E)** physical function during the follow-up period between the cognitive behavior therapy (CBT) and control groups. Std, standardized; iv, inverse variance; CI, confidence interval.

Analysis of a total of four RCTs with information about insomnia showed a medium treatment effect of CBT on severity of insomnia immediately after treatment (SMD = –0.65, 95% CI: –1.06 to –0.24, *P* = 0.002, *I*^2^ = 81%, four RCTs, 639 patients) (Supplementary Figure 2) (37, 42, 43, 47) with a consistent finding on sensitivity analysis. In the follow-up period, the beneficial effects of CBT on insomnia severity demonstrated a small treatment effect (SMD = –0.43, 95% CI: –0.85 to –0.01, *P* = 0.04, *I*^2^ = 82%, three RCTs, 571 patients), suggesting an association between CBT and a lower severity of insomnia (Figure 4A) (39, 42, 47). However, sensitivity analysis revealed an instability of the result.

#### 3.2.3. Sleep efficiency

Three studies provided the details regarding the impact of CBT on sleep efficiency immediately after treatment (35, 37, 42) while two RCTs reported this information in the follow-up period (39, 42). The use of CBT had a small treatment effect on sleep efficiency immediately after treatment (SMD = 0.32, 95% CI: 0.04 to 0.59, *P* = 0.03, *I*^2^ = 32%, three RCTs, 352 patients) (Figure 4B) (35, 37, 42) with an unstable finding on sensitivity analysis. However, there was a similar treatment effect on sleep efficiency between the two groups during the follow-up period (SMD = 0.07, 95% CI: –0.17 to 0.3, *P* = 0.58, two studies, 285 patients) (Supplementary Figure 3) (39, 42) with a consistent result on sensitivity analysis.

#### 3.2.4. Severity of depression symptom

The pooled results immediately after treatment and during follow-up period were available in five (35, 40, 44, 46, 47) and four RCTs (44–47), respectively. Immediately after intervention, there was comparable treatment effect of CBT in depression symptoms (SMD = –0.26, 95% CI: –0.58 to 0.06, *p* = 0.12, *I*^2^ = 75%, five studies, 735 patients) (Supplementary Figure 4) (35, 40, 44, 46, 47). During the follow-up period, there was a small treatment effect of CBT on depression severity (SMD = –0.39, 95% CI: –0.59 to –0.18, *p* = 0.0002, *I*^2^ = 48%, four RCTs, 791 patients) (Figure 4C) (44–47). Sensitivity analysis supported the stability of these two outcomes.

#### 3.2.5. Fatigue symptom and physical function

Regarding the severity of fatigue, there was similar treatment effect on this outcome immediately after treatment (SMD = –2.44, 95% CI:-6.53 to 1.65, *P* = 0.24, *I*^2^ = 100%, two RCTs, 511 patients) (Supplementary Figure 5) (38, 47) and during the follow-up period (SMD = –2.12, 95% CI:-5.53 to 1.28, *P* = 0.22, *I*^2^ = 99%, two RCTs, 464 patients) (Figure 4D) (38, 47). In respect of physical function, there was comparable treatment effect in the CBT group compared with the controls immediately after treatment (SMD = –0.11, 95% CI:-0.25 to 0.02, *P* = 0.09, *I*^2^ = 20%, five RCTs, 720 patients) (Supplementary Figure 6) (33, 36, 38, 41, 46) and during the follow-up period (SMD = –0.83, 95% CI:-1.87 to 0.22, *P* = 0.12, *I*^2^ = 98%, four RCTs, 801 patients) (Figure 4E) (34, 38, 45, 46). Sensitivity analysis of the outcomes of fatigue and physical function demonstrated unstable results regardless of the timing of evaluation (i.e., during immediate post-treatment or follow-up periods).

### 3.3. Meta-regression

Meta-regression analyses demonstrated no significant association of the included covariates (i.e., age, female ratio, sample size, and follow-up time) with the impact of CBT on pain severity (i.e., two-sided *p* > 0.05) (Table 2).

**TABLE 2:** Meta-regression for analysis.

	Covariates	Regression coefficient	Standard error	95% CI for coefficient	*Z*-value	*P*-value
Impact of CBT on pain relief immediately after treatment	Age (years)	0.0156	0.0923	–0.165 to 0.197	0.17	0.8659
	Female (%)	0.0391	0.0722	–0.102 to 0.181	0.54	0.5883
	Sample size	-0.0022	0.0041	–0.01 to 0.006	-0.54	0.5872
Impact of CBT on pain relief during follow-up	Age (years)	0.105	0.3311	–0.544 to 0.754	0.32	0.7511
	Female (%)	-0.0546	0.139	–0.327 to 0.218	-0.39	0.6946
	Sample size	-0.009	0.0227	–0.053 to 0.036	-0.39	0.6932
	Follow-up time (weeks)	0.0095	0.0601	–0.108 to 0.127	0.16	0.8746

CI, confidence interval.

## 4. Discussion

The results of the current studies had several striking clinical implications. Given that chronic pain associated with osteoarthritis is a major factor contributing to an impaired quality of life and socioeconomic burden (48–50), our results may suggest the effectiveness of psychological intervention as an alternative or complementary treatment strategy to the recommended pharmacological treatment (e.g., NSAIDs) (10, 26) in this setting. The lack of adverse side effects of medications may be another merit. To the best of our knowledge, this is the most comprehensive systematic review and meta-analysis focusing on the efficacy of CBT alone against osteoarthritis-associated symptoms with the inclusion of a recently published large-scale RCT of telephone CBT-I. The current meta-analysis of 15 RCTs showed an association of the implementation of CBT only with an improvement in the severity of insomnia and sleep efficiency immediately after intervention, but not in other symptoms including pain severity, depression, fatigue, and physical function in the immediate post-treatment period. During the follow-up period, we found that the use of CBT improved the severity of pain, insomnia, and depression. Nevertheless, sleep efficiency, fatigue, and physical function were not improved in the follow-up period.

In terms of the efficacy of CBT for osteoarthritis-related pain relief, the evidence reported in individual RCTs was mixed. The discrepancy may result from the use of different CBT approaches. For instance, instead of the application of CBT for pain, some studies used CBT for insomnia or CBT for depression. In this regard, previous published trials focusing on the effectiveness of pain coping skills training (PCST) in the population with osteoarthritis reported significant pain improvement during the immediate post-treatment period as well as during 12-month follow-ups, despite variation in intervention delivery (33, 38, 41). Regarding CBT for insomnia (CBT-I), the relevant trials also demonstrated mixed findings (35, 37, 39, 42, 47) probably attributable to the small sample sizes and less rigorous control groups in those reporting negative findings (6). On the other hand, another study demonstrated an improvement in osteoarthritis-related pain through internet CBT for depression at 3-month follow-up but not during the immediate post-treatment period (44). Nevertheless, the results of those trials need to be judiciously interpreted because of their inclusion of pain-related measurements mainly as secondary outcomes.

Our findings in patients with knee/hip osteoarthritis showed a medium treatment effect of CBT on pain severity during the follow-up period despite only a similar treatment effect immediately after intervention. The result is consistent with that of a prior review article that demonstrated the effectiveness of CBT for pain relief during long-term follow-up in the chronic pain population with fibromyalgia syndrome (16). On the other hand, different from our findings, another meta-analysis comprising participants with chronic non-cancer pain comorbid with insomnia revealed a correlation between CBT-I and short-term pain improvement but not in the follow-up period (51). Nevertheless, the use of CBT-I, a therapeutic tool primarily designed for insomnia, in that meta-analysis (51) may not accurately reflect the efficacy of the general CBT approach for chronic pain treatment. In contrast with previous systematic reviews that investigated the efficacy of CBT for a variety of chronic non-cancer pain (17, 51), one of the merits of the current study was our focusing on the knee/hip osteoarthritis population. Regarding the characteristics of our included studies, we found a tendency of increased utilization of internet- or telephone-based CBTs in the last decade compared to prior studies that mainly used face-to-face approaches. Technological evolution, which has overhauled the mode of communication in the recent decade, may be a possible explanation.

Focusing on the secondary outcomes, our results suggested small to medium treatment effect of CBTs on insomnia severity during both the immediate post-treatment and follow-up periods. A previous study has shown an association between sleep disturbance and osteoarthritis-induced pain (52). In addition, a recent systematic review demonstrated a correlation of the diagnosis of temporomandibular joint osteoarthritis with sleep quality and sleep disorders (e.g., obstructive sleep apnea) (53), further highlighting the adverse impact of osteoarthritis on the normal sleep cycle. Our findings were consistent with those of a prior meta-analysis (51) that demonstrated a significant and sustained therapeutic efficacy of CBT-I against insomnia in patients with chronic pain. It was noteworthy that the improvement in insomnia remained significant after the inclusion of a large-scale telephone CBT trial (47). However, our study showed a comparable treatment effect of CBT on sleep efficiency during follow-ups. In addition to the limited number of studies available for analysis, reliance on retrospective self-reported data (6) may introduce bias to the outcome. Despite the established effectiveness of CBT-I against comorbid insomnia from previous meta-analyses (54, 55), the current investigation was the first to focus on patients with knee/hip osteoarthritis.

Regarding the efficacy of CBT for depression among patients diagnosed with knee osteoarthritis, a previous trial including 300 participants failed to show significant treatment benefits immediately after CBT despite the demonstration of improvements at post-treatment one and six months (46). In contrast, another relatively small-scale trial on 69 individuals with knee osteoarthritis investigating the effectiveness of CBT against depression demonstrated significant improvement in depression during both immediate post-treatment and follow-up periods (44). Nevertheless, examination of the pooled evidence in the current study revealed a small treatment effect of CBT on depressive symptoms during follow-ups, while no benefit was noted in the immediate post-treatment period. The absence of a significant improvement during the immediate post-treatment period may be attributed to a variation in therapeutic protocols [e.g., group CBT (46) vs. internet CBT for depression (44)] across the included studies.

With respect to the other secondary outcomes, the current study revealed no significant benefit of CBT in the treatment of fatigue. Our finding was consistent with that of a previous meta-analysis focusing on the effectiveness of CBT-I for chronic pain with insomnia that showed no therapeutic effect against fatigue both during the immediate post-treatment and follow-up periods (51). Regarding physical function, the present investigation demonstrated no therapeutic benefit of CBT in both periods. Consistently, another meta-analysis investigating the use of pain coping skills training in patients with osteoarthritis also failed to reveal a positive impact of CBT on physical function (56). The small numbers of studies available for analysis may contribute to the lack of significant treatment benefits regarding the two outcomes in the current study. While the outcome on fatigue was derived from only two studies, the result on physical function was based on five and four trials for the immediate post-treatment and follow-up periods, respectively. Therefore, further studies are warranted to verify our findings.

There are several limitations in the current study. First, the high heterogeneities across our included studies attributable to the diversity in therapeutic protocols, modes of delivery, therapist (e.g., trained nurse vs. physical therapist) as well as the number of treatment sessions and duration (Supplementary Table 3) may introduce bias to our outcomes. Besides, the use of different pain assessment tools (e.g., AIMS pain subscale, WOMAC pain subscale, and VAS scale), the recruitment of a mixed population of patients diagnosed with knee or hip osteoarthritis, and variations in follow-up periods may be potential sources of bias. Second, publication bias may arise from our inclusion of English literature for analysis. Third, the risk of bias assessment in the current meta-analysis showed that the overall risk of bias was high and of some concerns in two and 13 studies, respectively. The risk of bias may mostly be attributed to the randomization process, deviations from intended intervention, and missing outcome data. Therefore, our results may be biased due to the poor quality of those studies. Finally, despite our demonstration of the effectiveness of CBT against pain, insomnia, and depression during the follow-up periods, the selection of different cut-off points and follow-up periods as well as the merging of results from different time points (36, 38) across the included studies precluded our elucidation of the durability of its efficacy.

## 5. Conclusion

This systematic review and meta-analysis showed the efficacy of CBTs against osteoarthritis-related pain, insomnia, and depression during the follow-up period but without significant benefit in the treatment of sleep efficiency, fatigue, and physical function. Our results may suggest the durability of CBT-associated treatment effects, supporting its role as a potential promising alternative or complementary intervention for patients with knee/hip osteoarthritis, especially against pain and insomnia. Nevertheless, the high heterogeneity across the included studies warrants future large-scale investigations to verify our findings.

## Data availability statement

The original contributions presented in this study are included in the article/Supplementary material, further inquiries can be directed to the corresponding author.

## Author contributions

H-ML and P-SH: conceptualization. N-CC: methodology. C-HT: software. N-CC and W-FK: formal analysis. N-CC and H-ML: data curation. H-ML, P-SH, and K-CH: writing—original draft preparation. K-CH and Y-LL: writing—review and editing. All authors have read and agreed to the published version of the manuscript.
