# Supplementary material for: Impact of cognitive behavior therapy on osteoarthritis-associated pain, insomnia, depression, fatigue, and physical function in patients with knee/hip osteoarthritis: A systematic review and meta-analysis of randomized controlled trials
Source: Front Med (Lausanne). 2023 Jan 6;9:1083095. doi: 10.3389/fmed.2022.1083095 (PMC9853196; doi:10.3389/fmed.2022.1083095)

**Supplemental Table 1.** Search strategies for databases

| Database |  | Search syntax |
| --- | --- | --- |
| **MEDLINE (Ovid)** | #1 | (“Osteoarthritis” OR “OA knee” OR “OA hip” OR “knee osteoarthritis” OR “hip osteoarthritis”).mp |
|  | #2 | exp "osteoarthritis"/ OR exp "osteoarthritis, hip"/ OR exp "osteoarthritis, knee"/ |
|  | #3 | (“Cognitive behavioral therap*” OR “Cognitive Psychotherap*” OR “CBT” OR “behavior*therap*” OR “cognitive-behavioral therapy” OR “cognitive therap*” OR “pain coping skill training” OR “behavior graded activity”).mp |
|  | #4 | exp "Cognitive behavioral therapy"/ |
|  | #5 | (1 OR 2) AND (3 OR 4) |
|  | #6 | #5 AND (randomized controlled trial.pt. or controlled clinical trial.pt. or randomi*ed.ab. or placebo.ab. or drug therapy.fs. or randomly.ab. or trial.ab. or groups.ab. not (exp animals/ not humans.sh.)) |
| **Embase (Ovid)** | #1 | (“Osteoarthritis” OR “OA knee” OR “OA hip” OR “knee osteoarthritis” OR “hip osteoarthritis”).mp |
|  | #2 | Exp "osteoarthritis"/ OR exp "osteoarthritis, hip"/ OR exp "osteoarthritis, knee"/ |
|  | #3 | (“Cognitive behavioral therap*” OR “Cognitive Psychotherap*” OR “CBT” OR “behavior*therap*” OR “cognitive-behavioral therapy” OR “cognitive therap*” OR “pain coping skill training” OR “behavior graded activity”).mp |
|  | #4 | Exp "Cognitive behavioral therapy"/ |
|  | #5 | (1 OR 2) AND (3 OR 4) |
|  | #6 | #5 AND ('crossover procedure':de OR 'double-blind procedure':de OR 'randomized controlled trial':de OR 'single-blind procedure':de OR (random* OR factorial* OR crossover* OR cross NEXT/1 over* OR placebo* OR doubl* NEAR/1 blind* OR singl* NEAR/1 blind* OR assign* OR allocat* OR volunteer*):de,ab,ti) |
| **Cochrane CENTRAL** | #1 | (“Osteoarthritis” OR “OA knee” OR “OA hip” OR “knee osteoarthritis” OR “hip osteoarthritis”):ti,ab,kw |
|  | #2 | [mh "osteoarthritis"] OR [mh "osteoarthritis, hip"] OR [mh "osteoarthritis, knee"] |
|  | #3 | (“Cognitive behavioral therap*” OR “Cognitive Psychotherap*” OR “CBT” OR “behavior*therap*” OR “cognitive-behavioral therapy” OR “cognitive therap*” OR “pain coping skill training” OR “behavior graded activity”):ti,ab,kw |
|  | #4 | [mh "Cognitive behavioral therapy"] |
|  | #5 | (#1 OR #2) AND (#3 OR #4) |
| **Google**  **scholar** |  | A hand-search strategy was used to identify the related studies, followed by using a forward snowballing strategy to retrieve all potentially eligible articles. |

**Supplementary Table 2:** Method for osteoarthritis diagnosis

| **Author (Year) (Country)** | **Method for osteoarthritis diagnosis** |
| --- | --- |
| Keefe et al. (1990) (USA) | Knee OA (100%) Dx: Medical evaluation and radiographic examination |
| Veenhof et al. (2006) (The Netherland) | Knee OA (65%), Hip OA (40%), Both (10%) Dx: Medical evaluation by physician; radiographic assessment |
| Vitiello et al. (2009) (USA) | Dx: Physician-confirmed; radiograph or MRI |
| Somers et al. (2012) (USA) | Knee OA (100%) Dx: Medical evaluation by physician; Radiographic assessment (ACR knee OA classification criteria) |
| Vitiello et al. (2013) (USA) | Dx: Medical care for OA in the prior 3 years; Significant OA pain as defined by Grade II, III, or IV pain on the GCPS |
| McCurry et al. (2014) (USA) | Dx: Medical care for OA in the prior 3 years; Significant OA pain as defined by Grade II, III, or IV pain on the GCPS |
| Broderick et al. (2014) (USA) | Knee OA (77.4%), Hip OA (22.6%) Dx: Physician-confirmed diagnosis; Usual pain ≥ 4 on a 10-point scale for a duration of at least 6 months |
| Helminen et al (2015) (Finland) | Knee OA (100%) Dx: Clinical symptoms and radiographic grading (Kellgren-Lawrence 2-4) |
| Smith et al. (2015) (USA) | Knee OA (100%) Dx: Rheumatologist medical evaluation; radiographic evidence; knee pain ratings (ACR knee OA classification criteria) |
| Rini et al. (2015) (USA) | Knee OA (35.3%), Hip OA (12.3%), Both (52.2%) Dx: Physician-confirmed and radiographic grading (KL grade ≥2, with pain in the affected joint) (ACR knee OA classification criteria) |
| Heffner et al. (2018) (USA) | Knee OA (100%) Dx: Physician-confirmed and radiographic evidence |
| O'moore et al. (2018) (Australia) | Knee OA (100%) Dx: radiographic criteria; knee pain on most days (ACR knee OA classification criteria) |
| Allen et al. (2019) (USA) | Knee OA (79.3%), Hip OA (10.7%), Both (10%) Dx: Diagnosis of OA based on previous medical evaluation.; self-report OA pain at most days for the past month |
| Foo et al. (2020) (Malaysia) | Knee OA (100%) Dx: Medical evaluation by physician and radiographic evidence (KL classification of grade 2 or higher) |
| McCurry et al. (2021) (USA) | Dx: Diagnosis of OA in the 3 years prior to screening by medical evaluation |

OA, Osteoarthritis

**Supplementary Table 3**: The CBT protocol and components of the included studies

| **Author (Year)** | **Model of CBT** | **Delivery of intervention** | **Duration** | **Frequency** | **Session descriptions** |
| --- | --- | --- | --- | --- | --- |
| Keefe et al. (1990) | PCST | Group, 6-9 people | 10-week sessions, each 90 minutes | Every week | Cognitive and behavioral coping methods; relaxation, imagery, and distraction techniques; activity-rest cycling and pleasant activity scheduling; cognitive restructuring |
| Veenhof et al. (2006) | BGA | Individual, face-to-face | 12-week period with a maximum of 18 sessions, followed by 5 preset booster moments with a maximum of 7 sessions | Every week | BGA protocol and included written materials (education messages, activity diaries, performance charts) |
| Vitiello et al. (2009) | CBT-I, by Morin’s protocol | Group, 4-8 people | 8-week sessions, each 2 hours | Every week | Stimulus control; sleep restriction; cognitive restructuring; relaxation training; sleep-hygiene education |
| Somers et al. (2012) | PCST+BWM PCST-only | Group | 24-week sessions, each 60 minutes | Every week (the first 12 weeks),  every 2 weeks (last 12 weeks) | Pain coping strategies; attention diversion methods: relaxation, imagery, and distraction; activity-rest cycling and pleasant activity scheduling; cognitive-restructuring; methods for coping with common problems |
| Vitiello et al. (2013) | CBT-P/CBT-PI | Group | 6-week sessions, each 90 mins | Every week | CBT-P: pain education, physical activation, goal setting, relaxation, activity pacing, guided imagery, and cognitive restructuring. CBT-PI: standard components of CBT for insomnia (sleep hygiene education, stimulus control, sleep restriction, and daily sleep monitoring) added to the CBT-P intervention. |
| McCurry et al. (2014) | CBT-P/CBT-PI | Group | 6-week sessions, each 90 mins | Every week | As The Lifestyles trial protocol |
| Broderick et al. (2014) | PCST | Individual Face to face / telephone | Total 10 sessions, each 30-45 minutes | Every week | Relaxation response; attention diversion techniques; altering activity and rest patterns as a way of increasing activity level; reducing negative pain-related thoughts and emotions |
| Helminen et al (2015) | CBT, presented by Linton | Group, 7-13 people | 6-week sessions, each 2 hours | Every week | Introduction (15minutes); lecture (knowledge and insight, max 15minutes); problem solving (in pairs/teams, 15−20minutes); skills training (15−20minutes); homework assignments (15minutes); a résumé (feedback) of the session (15 minutes). |
| Smith et al. (2015) | CBT-I | Group | Total 8 sessions, each 45 mins | Every week | Sleep restriction therapy; stimulus control therapy; cognitive therapy for insomnia; sleep hygiene education |
| Rini et al. (2015) | Interne PCST (PainCOACH) | Internet-based | Total 8 modules, each 35-45 minutes | Every week | Progressive muscle relaxation; mini-practices; activity/rest cycling; pleasant activity scheduling; negative automatic thoughts; coping thoughts; pleasant imagery and other distraction techniques; problem solving; monitoring for maintenance |
| Heffner et al. (2018) | CBT-I | Not presented | 6-week sessions | Every week | Not presented |
| O'moore et al. (2018) | Internet-based CBT, for MDD | Internet-based | 6 online lessons for 10 weeks | Individual-based | 6 online lessons, with regular homework assignments and access to supplementary resources |
| Allen et al. (2019) | PCST | Telephone-based | 11-week sessions, each 30-45 minutes | Every week | Introduction; mini-relaxation practices; managing unhelpful mood; activity pacing; pleasant activities; pleasant imagery; physical activity; weight management; skills review and problem solving; relapse prevention and maintenance |
| Foo et al. (2020) | Modified CBT, for knee pain | Group, 8-12 people | Total 3 sessions, each 2.5 hours | Every 2 weeks | First (overview); second (time-based pacing); third (Sleep quality/relapse prevention/dealing with flare-ups) |
| McCurry et al. (2021) | CBT-I | Telephone  manual-based | 6 sessions over 8 weeks, each 20-30 minutes | Every week | In-bed restriction plan; stimulus control instructions; sleep hygiene education; cognitive strategies |

Abbreviations: BGA, Behavior Graded Activity; BWM, lifestyle behavioral weight management; MDD, Major Depression Disorder; CBT-I, Cognitive Behavior Therapy for Insomnia; CBT-P, Cognitive Behavior Therapy for Pain; CBT-PI, Cognitive Behavior Therapy for Pain and Insomnia; MDD, Major Depression Disorder; PCST, Pain Coping Skills Training

| **Supplemental Table 4.** Overall quality of evidence according to GRADE assessment^a^ | | | | | | | |
| --- | --- | --- | --- | --- | --- | --- | --- |
|  |  | Certainty Assessment | | | | | |
|  | No. of Patients/Studies | Risk of Bias | Inconsistency | Indirectness | Imprecision | Publication Bias | Overall Certainty of Evidence^b^ |
| Pain Severity at post-treatment | 1557/11 | Not serious | Serious^c^ | Not serious | Not serious | None | Moderate |
| Pain Severity at follow-up | 1447/8 | Not serious | Serious^c^ | Not serious | Not serious | undetected | Moderate |
| Insomnia Severity at post-treatment | 639/4 | Not serious | Serious^c^ | Not serious | Not serious | undetected | Moderate |
| Insomnia Severity at follow-up | 571/3 | Not serious | Serious^c^ | Not serious | Not serious | undetected | Moderate |
| Sleep Efficiency at post-treatment | 352/3 | Not serious | Not serious | Not serious | Serious^d^ | undetected | Moderate |
| Sleep Efficiency at follow-up | 285/2 | Not serious | Not serious | Not serious | Serious^d^ | undetected | Moderate |
| Depression at post-treatment | 735/5 | Not serious | Serious^c^ | Not serious | Not serious | undetected | Moderate |
| Depression at follow-up | 791/4 | Not serious | Not serious | Not serious | Not serious | undetected | High |
| Fatigue at post-treatment | 511/2 | Not serious | Serious^c^ | Not serious | Not serious | undetected | Moderate |
| Fatigue at follow-up | 464/2 | Not serious | Serious^c^ | Not serious | Not serious | undetected | Moderate |
| Physical Function at post-treatment | 720/5 | Not serious | Not serious | Not serious | Not serious | undetected | High |
| Physical Function at follow-up | 801/4 | Not serious | Serious^c^ | Not serious | Not serious | undetected | Moderate |
| ^a^GRADE, Grading of Recommendations Assessment, Development and Evaluation. ^b^Overall certainty of evidence: very low, low, moderate, and High. ^c^Unexplained high heterogeneity. ^d^Did not meet optimal information size criterion | | | | | | | |

**Supplementary Figure 1.** Publication bias for pain severity immediately after treatment

**
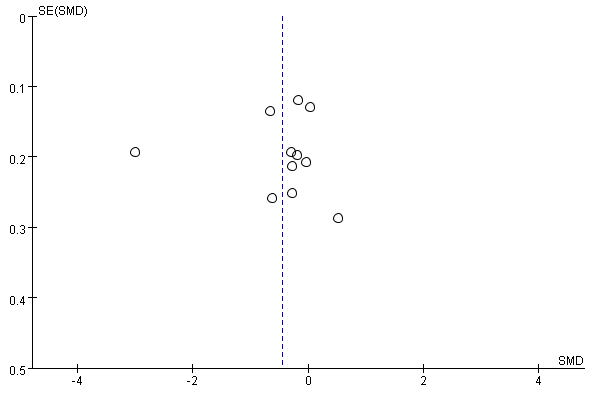
**

**Supplementary Figure 2.** Forest plot comparing insomnia severity immediately after treatment between the cognitive behavior therapy (CBT) and control groups. Std: standardized; iv: inverse variance; CI: confidence interval


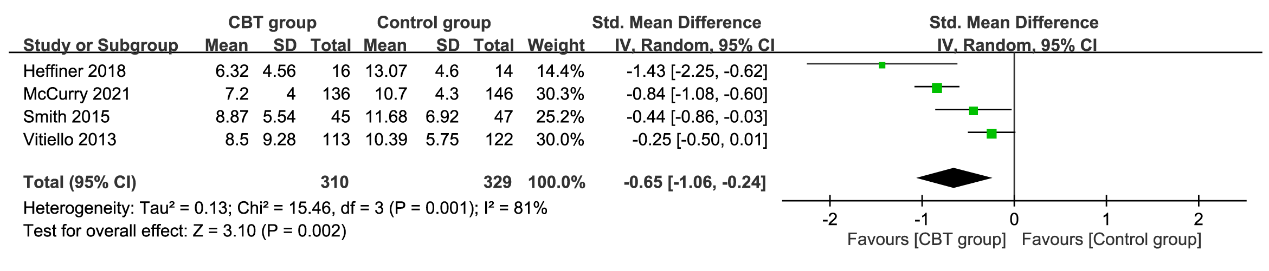


**Supplementary Figure 3.** Forest plot comparing sleep efficiency immediately after treatment between the cognitive behavior therapy (CBT) and control groups. Std: standardized; iv: inverse variance; CI: confidence interval


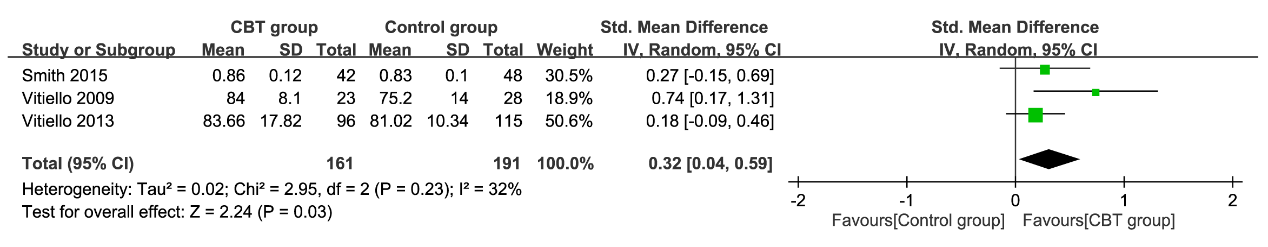


**Supplementary Figure 4.** Forest plot comparing depression severity immediately after intervention between the cognitive behavior therapy (CBT) and control groups. Std: standardized; iv: inverse variance; CI: confidence interval.


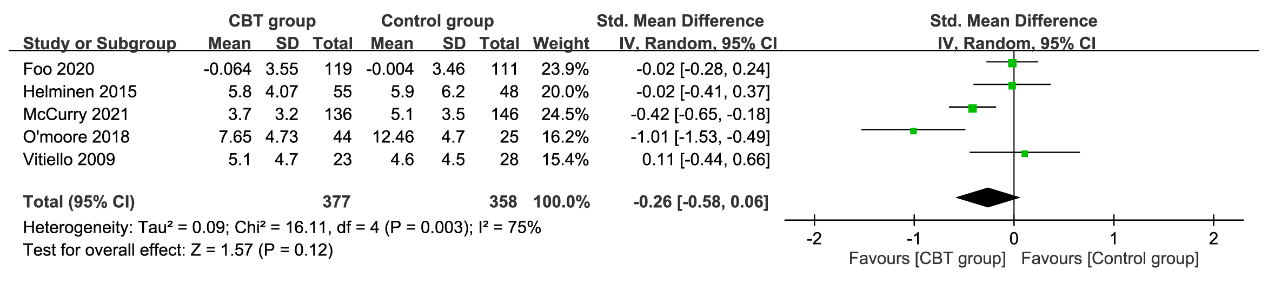


**Supplementary Figure 5** Forest plot comparing fatigue severity immediately after treatment between the cognitive behavior therapy (CBT) and control groups. Std: standardized; iv: inverse variance; CI: confidence interval.


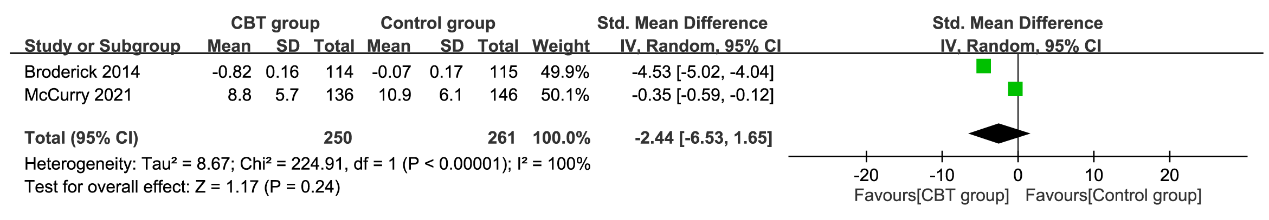


**Supplementary Figure 6.** Forest plot comparing physical function immediately after treatment between the cognitive behavior therapy (CBT) and control groups. Std: standardized; iv: inverse variance; CI: confidence interval.


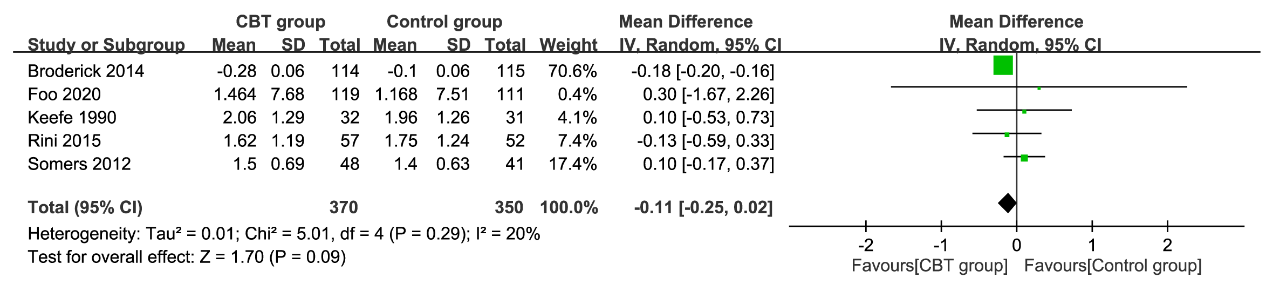

Supplement: Supplementary file 1 [file Data_Sheet_1.docx]
